# Connections between Endometrial Health Status, Fatty Liver and Expression of Endocannabinoid System Genes in Endometrium of Postpartum Dairy Cows

**DOI:** 10.3390/ijms25179187

**Published:** 2024-08-24

**Authors:** Zuzanna Polak, Milena Krupa, Joanna Sadowska, Paweł Brym, Maciej Ślebioda, Andrzej Jurczak, Dominika Grzybowska, Dawid Tobolski

**Affiliations:** 1 Department of Epizootiology, Faculty of Veterinary Medicine, University of Warmia and Mazury in Olsztyn, Oczapowskiego 13, 10-718 Olsztyn, Poland; zuzanna.polak@uwm.edu.pl; 2 Department of Animal Reproduction with Clinic, Faculty of Veterinary Medicine, University of Warmia and Mazury in Olsztyn, Oczapowskiego 14, 10-719 Olsztyn, Poland; milena.krupa@uwm.edu.pl (M.K.); maciej.slebioda@uem.edu.pl (M.Ś.); andrzej.jurczak@uwm.edu.pl (A.J.); 3 Department of Animal Genetics, Faculty of Animal Bioengineering, University of Warmia and Mazury in Olsztyn, Oczapowskiego 5, 10-719 Olsztyn, Poland; joanna.sadowska@uwm.edu.pl (J.S.); pawbrym@uwm.edu.pl (P.B.); 4 Department of Internal Medicine with Clinic, Faculty of Veterinary Medicine, University of Warmia and Mazury in Olsztyn, Oczapowskiego 14, 10-719 Olsztyn, Poland; dominika.wysocka@uwm.edu.pl

**Keywords:** dairy cows, endocannabinoid system, endometrial biopsy, endometritis, fatty liver, gene expression

## Abstract

The endocannabinoid system (ECS) plays a crucial role in reproductive health, but its function in postpartum dairy cows remains poorly understood. This study investigated the expression patterns of ECS-related genes in the endometrium of postpartum dairy cows and their associations with endometrial health and the presence of fatty liver. Endometrial biopsies were collected from 22 Holstein Friesian cows at 4 and 7 weeks postpartum. Gene expression was analyzed using RT-qPCR, focusing on key ECS components including *CNR2*, *MGLL*, *FAAH1*, *NAAA*, *NAPEPLD*, *PADI4* and *PTGDS.* The results reveal dynamic changes in ECS gene expression associated with endometritis and fatty liver. *MGLL* expression was significantly upregulated in cows with endometritis at 7 weeks postpartum, while *NAAA* expression was consistently downregulated in cows with fatty liver. *CNR2* showed a time-dependent pattern in endometritis, and *PTGDS* expression was elevated in clinical endometritis at 4 weeks postpartum. The presence of fatty liver was associated with altered expression patterns of several ECS genes, suggesting a link between metabolic stress and endometrial ECS function. These findings indicate a potential role for the ECS in postpartum uterine health and recovery, offering new insights into the molecular mechanisms underlying reproductive disorders in dairy cows and paving the way for novel therapeutic approaches.

## 1. Introduction

The endocannabinoid system (ECS) is a complex lipid-based signaling mechanism that plays a critical role in maintaining neural and metabolic homeostasis, while also exerting significant influence on the female reproductive system [1,2]. This intricate system comprises endocannabinoids (eCBs), cannabinoid receptors and enzymes responsible for eCB synthesis and degradation. The primary endocannabinoids, N-arachidonoylethanolamide (anandamide, AEA) and 2-arachidonoylglycerol (2-AG), interact with the canonical cannabinoid receptors CB1 (gene *CNR1*) and CB2 (gene *CNR2*) [3].

**Figure 1:**
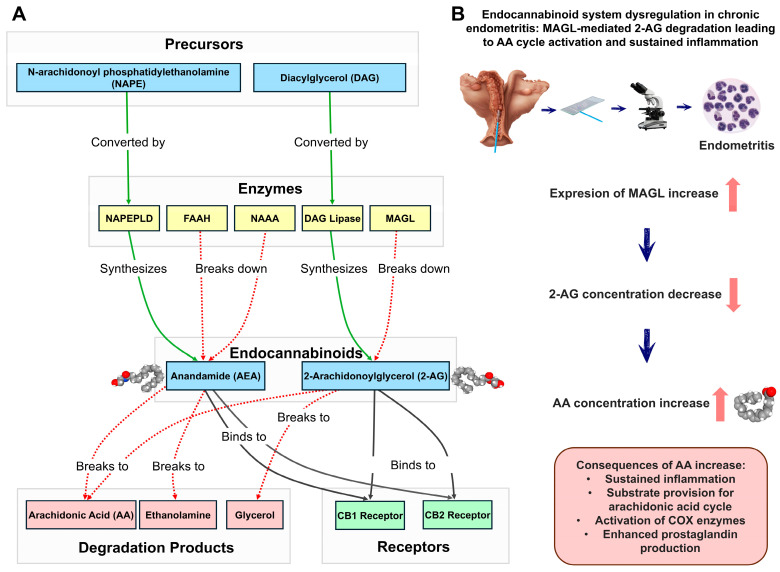
Schematic representation of the endocannabinoid system and its dysregulation in endometritis. Panel (**A**) illustrates the endocannabinoid synthesis and degradation pathways. Precursors N-arachidonoyl phosphatidylethanolamine (NAPE) and Diacylglycerol (DAG) are converted by enzymes NAPEPLD and DAG Lipase, respectively, to produce endocannabinoids Anandamide (AEA) and 2-Arachidonoylglycerol (2-AG). These endocannabinoids bind to CB1 and CB2 receptors. Degradation enzymes FAAH, NAAA and MAGL break down endocannabinoids into their constituent parts. Panel (**B**) depicts the hypothesized mechanism of MAGL-mediated 2-AG degradation leading to sustained inflammation in endometritis.

The AEA is synthesized from N-arachidonoyl phosphatidylethanolamine (NAPE) by NAPE phospholipase D (NAPEPLD, gene *NAPEPLD*), while 2-AG is synthesized from diacylglycerol (DAG) by DAG lipase. Their breakdown is mediated by enzymes including monoacylglycerol lipase (MAGL, gene *MGLL*), fatty acid amino hydrolase (FAAH, gene *FAAH1*) and N-acylethanolamine-hydrolyzing acid amidase (NAAA, gene *NAAA*) [4,5]. These endocannabinoids play crucial roles in various physiological processes, including mood regulation, appetite control, pain sensation and memory. In reproduction, they are particularly important for fertilization, embryo implantation, pregnancy maintenance, and parturition, with their delicate balance being critical for successful reproductive outcomes [6,7] (Figure 1A).

AEA primarily binds to CB1 receptors in the central nervous system and adipose tissue, influencing neural functions, while its interaction with CB2 receptors on immune cells modulates immune response and inflammation. 2-AG, on the other hand, binds with similar affinity to both CB1 and CB2 receptors, exerting substantial effects on neural processes, pain perception and immune modulation. The activity of MAGL in reproductive health varies across different phases of the estrous cycle and can significantly affect local concentrations of 2-AG in the endometrium, impacting processes such as implantation and inflammation [7,8,9].

The roles of AEA and 2-AG in inflammation are complex and context-dependent. While they can exert anti-inflammatory effects through CB2 receptor interactions, the degradation of 2-AG by MAGL produces arachidonic acid (AA), a precursor for pro-inflammatory eicosanoids. This highlights the delicate balance maintained by the ECS in inflammation, which is particularly relevant in the context of reproductive health [9,10] (Figure 1B).

Other genes that encode proteins, which interact with or influence the ECS, contribute to its complex regulatory network. The Peptidyl Arginine Deiminase 4 (PADI4, gene *PADI4*) plays a role in inflammation and immune response, particularly in granulocyte and macrophage development. Its activation is associated with neutrophil extracellular trap (NET) formation [11], crucial in the innate immune response of the postpartum uterus in dairy cows. However, uncontrolled NET formation can be detrimental to the reproductive tract [12]. The Prostaglandin D2 Synthase (PTGDS, gene *PTGDS*) catalyzes the conversion of prostaglandin H2 (PGH2) into prostaglandin D2 (PGD2), involved in neuromodulation, smooth muscle function and anandamide metabolism. This interplay between the ECS and prostaglandin synthesis pathways further illustrates the intricate network of signaling molecules in reproductive processes [13,14].

While much of our understanding of the ECS comes from human studies, recent research has explored its role in various animal species, including dairy cows. Studies have focused on the ECS’s involvement in adipose tissue function, metabolic profiles during the post-parturition period and its role in the bovine endometrium [9,15,16,17]. These investigations have revealed the potential importance of the ECS in the reproductive health and efficiency of dairy cows.

Understanding uterine health in dairy cows is crucial due to its economic and welfare implications. Subclinical endometritis (SE), often resulting from maladaptation during the transition period and metabolic stress, presents a particular challenge with no efficient treatment methods currently available [18,19]. This highlights the need for novel approaches to managing uterine diseases in dairy cows, which could potentially be developed through a deeper understanding of the ECS’s role in endometrial health.

The metabolic changes during the postpartum period in dairy cows are intricately linked to the status of liver health. One of the most prevalent metabolic disorders affecting this organ is fatty liver, also known as fat cow syndrome, which manifests when there is a disruption in the balance between lipid uptake, oxidation and secretion. This condition arises when an excessive amount of lipids accumulates in the liver, leading to significant metabolic disturbances and compromising overall hepatic function [20]. Decreased reproductive performance can also be observed during hepatic malfunction. Postpartum cows suffering from endometritis often exhibit reduced concentrations of albumin and magnesium in their blood, which may be indicative of impaired liver function [21]. Key metabolic parameters, such as glucose and BHBA, which are closely associated with energy status and have been extensively studied, can serve as valuable risk factors for both liver malfunction as well as endometritis [22]. These interconnections underscore the importance of considering reproductive health and metabolic status, particularly liver condition, as interconnected factors when developing strategies for managing the transition period in dairy cows.

The present study aims to explore the expression patterns of endocannabinoid system (ECS) genes in the endometrium of postpartum dairy cows and their potential associations with endometrial health. To achieve this, we will quantify and compare the expression levels of ECS-related genes in the endometrium of healthy cows and those with endometritis at various postpartum stages, while also investigating the potential influence of fatty liver presence. The analysis of the ECS in the bovine endometrium during the postpartum period has the potential to inform the development of novel diagnostic tools, therapeutic interventions or management strategies to improve reproductive efficiency and overall health in dairy cow herds. Moreover, it contributes to our broader understanding of comparative reproductive biology across species, highlighting the complex interplay between the ECS, inflammatory processes and reproductive functions in mammals.

## 2. Results

### 2.1. Dynamics of Endometrial Gene Expression in Postpartum Dairy Cows with Endometritis

The gene expression analysis of endometrial biopsies from postpartum dairy cows revealed distinct patterns related to endometrial health. Healthy cows sampled at 4 weeks postpartum (H4) served as the control group, providing a baseline for comparisons. In the context of endometritis, several genes showed notable changes in expression patterns over time.

*CNR2* expression exhibited a time-dependent pattern in cows with endometritis. At 4 weeks postpartum, both subclinical (SE4) and clinical (CE4) endometritis groups showed decreased expression compared to healthy controls, with fold changes of 0.55 ± 0.30 (*p* = 0.247) and 0.66 ± 0.62 (*p* = 0.571), respectively. However, by 7 weeks postpartum, this trend reversed, with clinical endometritis cows (CE7) showing the highest increase in *CNR2* expression (FC = 1.72 ± 0.91, *p* = 0.436).

**Figure 2:**
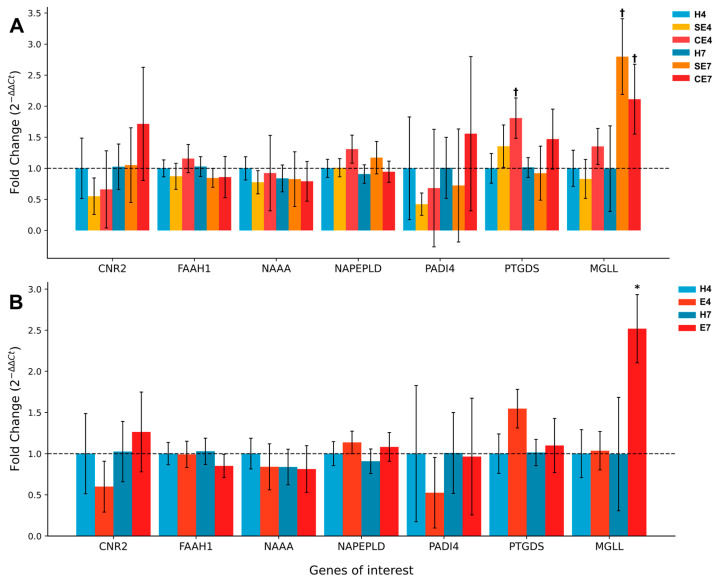
Relative expression of endocannabinoid system-related genes in the endometrium of postpartum dairy cows. Panel (**A**) shows gene expression in healthy cows (H), cows with subclinical endometritis (SE) and cows with clinical endometritis (CE) at 4 and 7 weeks postpartum. Panel (**B**) presents gene expression in healthy cows (H) versus cows with endometritis (E, combining subclinical and clinical cases) at 4 and 7 weeks postpartum. The y-axis represents fold change (2^−ΔΔCt^) relative to healthy cows at 4 weeks postpartum (H4). Error bars indicate standard error of the mean. Genes analyzed include cannabinoid receptor 2 (*CNR2*), fatty acid amide hydrolase (*FAAH1*), N-acylethanolamine acid amidase (*NAAA*), N-acyl phosphatidylethanolamine phospholipase D (*NAPEPLD*), peptidyl arginine deiminase 4 (*PADI4*), prostaglandin D2 synthase (*PTGDS*) and monoacylglycerol lipase (*MGLL*). Significant differences (*p* < 0.05) are indicated by asterisks (*), while statistical tendencies (0.05 ≤ *p* < 0.1) are marked with a cross (†).

*MGLL* expression demonstrated the most significant change among all genes studied in relation to endometritis. Cows with endometritis at 7 weeks postpartum showed a marked increase in *MGLL* expression. When subclinical and clinical endometritis groups were combined, the endometritis group at 7 weeks postpartum (E7) exhibited a significant increase with a fold change of 2.52 ± 0.41 (*p* = 0.023) (Figure 2).

*PTGDS* expression also showed an interesting pattern in relation to endometritis. Both subclinical (SE4) and clinical (CE4) endometritis groups displayed increased *PTGDS* expression at 4 weeks postpartum, with fold changes of 1.36 ± 0.34 and 1.81 ± 0.32 (*p* = 0.070), respectively. However, this elevation was not maintained at 7 weeks postpartum.

### 2.2. Impact of Fatty Liver on Endocannabinoid System-Related Gene Expression in Bovine Endometrium

In the analysis of fatty liver impact, several genes demonstrated notable changes in expression. *NAAA* expression showed a consistent downregulation in the presence of fatty liver. At 7 weeks postpartum, cows with fatty liver (FL7) exhibited a significant decrease in *NAAA* expression with a fold change of 0.62 ± 0.23 (*p* = 0.035). A similar trend was observed at 4 weeks postpartum (FL4, FC = 0.68 ± 0.17, *p* = 0.052), suggesting a persistent effect of fatty liver on *NAAA* expression.

*PADI4* expression tended to be lower in cows with fatty liver, particularly at 4 weeks postpartum (FL4), with a fold change of 0.44 ± 0.55 (*p* = 0.071). Although this change did not reach statistical significance, it indicates a potential early response of *PADI4* to the presence of fatty liver. The effect was less pronounced at 7 weeks postpartum, suggesting a possible adaptation over time.

**Figure 3:**
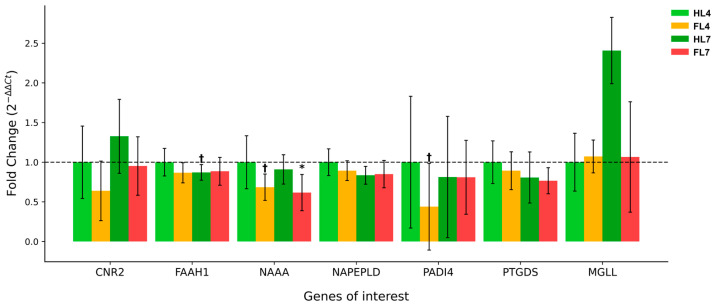
Relative expression of endocannabinoid system-related genes in the endometrium of postpartum dairy cows with and without fatty liver. The graph shows gene expression in healthy liver cows (HL) and cows with fatty liver (FL) at 4 and 7 weeks postpartum. The y-axis represents fold change (2^−ΔΔCt^) relative to healthy cows at 4 weeks postpartum (HL4). Error bars indicate standard error of the mean. Genes analyzed include cannabinoid receptor 2 (*CNR2*), fatty acid amide hydrolase (*FAAH1*), N-acylethanolamine acid amidase (*NAAA*), N-acyl phosphatidylethanolamine phospholipase D (*NAPEPLD*), peptidyl arginine deiminase 4 (*PADI4*), prostaglandin D2 synthase (*PTGDS*) and monoacylglycerol lipase (*MGLL*). Significant differences (*p* < 0.05) are indicated by asterisks (*), while statistical tendencies (0.05 ≤ *p* < 0.1) are marked with a cross (†).

*CNR2* expression in cows with fatty liver showed an interesting temporal pattern. At 4 weeks postpartum, there was a decrease in expression (FL4, FC = 0.64 ± 0.38, *p* = 0.142), while at 7 weeks, healthy cows showed an increase (HL7, FC = 1.33 ± 0.47, *p* = 0.495) that was not mirrored in the fatty liver group (FL7, FC = 0.95 ± 0.37, *p* = 0.868). This differential response suggests that fatty liver may interfere with the normal temporal regulation of *CNR2* expression in the postpartum period (Figure 3).

Other genes, including *FAAH1* and *NAPEPLD*, showed various changes in expression under different conditions, but these changes were generally less pronounced and did not reach statistical significance. *FAAH1* and *NAPEPLD* showed a trend toward decreased expression in the presence of fatty liver at both time points.

## 3. Discussion

Our study reveals variations in the expression of endocannabinoid system (ECS) genes in the endometrium of postpartum dairy cows, providing novel insights into the complex relationship between ECS, endometrial health and the presence of fatty liver. The observed expression patterns under different health conditions and postpartum stages enhance our understanding of the ECS’s role in bovine reproductive health and offer potential avenues for therapeutic interventions.

The expression profiles of *CNR2*, *FAAH1*, *NAAA*, *NAPEPLD*, *PADI4*, *PTGDS* and *MGLL* in endometrial biopsies from healthy cows and those with subclinical and clinical endometritis at 4 and 7 weeks postpartum revealed notable differences, although many did not reach statistical significance. This may be attributed to the inherent biological variability among individual animals and the complex nature of endometrial physiology during the postpartum period. Nevertheless, the observed trends provide valuable insights into the dynamic regulation of the ECS in response to endometrial inflammation and metabolic challenges.

One of the most interesting findings was the consistently higher expression of *CNR2* in cows with endometritis at 7 weeks postpartum compared to 4 weeks. This time-dependent increase suggests a potential adaptive response of the ECS to persistent endometrial inflammation. The upregulation of *CNR2*, which encodes the CB2 receptor, may represent a compensatory mechanism aimed at mitigating inflammation, as CB2 receptor activation is known to exert anti-inflammatory effects. This interpretation is supported by previous studies in other species, which have demonstrated increased CB2 receptor expression in inflamed tissues [23]. The temporal nature of this change highlights the importance of considering the dynamic postpartum environment when studying endometrial health in dairy cows.

The significant upregulation of *MGLL* in cows with endometritis at 7 weeks postpartum is a critical finding with potential implications for the persistence of endometrial inflammation. *MGLL* degrades 2-arachidonoylglycerol (2-AG), an endocannabinoid with potent anti-inflammatory properties. The increased *MGLL* expression we observed could lead to reduced 2-AG levels and, consequently, elevated levels of pro-inflammatory arachidonic acid derivatives [8]. This shift in the balance between anti-inflammatory and pro-inflammatory mediators may contribute to the chronic nature of endometritis in some cows. Our findings align with those of Dirandeh et al. [9], who reported altered expression of ECS components in adipose tissue of periparturient cows, suggesting a systemic impact of metabolic stress on ECS function.

The varied expression levels of FAAH1 and NAAA, enzymes involved in the degradation of anandamide (AEA), across different conditions provide further evidence of the ECS’s complex regulation in the postpartum period. The decreased expression of these enzymes in cows with endometritis at 4 weeks postpartum suggests a potential mechanism for increasing local AEA levels. Elevated AEA could serve to modulate inflammatory responses, as this endocannabinoid has been shown to have both pro- and anti-inflammatory effects depending on the cellular context and receptor activation [24]. The observation that *FAAH1* expression in endometritis cows approximated that of healthy cows by 7 weeks postpartum may indicate a shift toward the resolution of inflammation, although this hypothesis requires further investigation.

The study by Bonsale et al. [25] provides further corroboration for our findings. They reported that cows defined as subclinical endometritis/with endometrial inflammation exhibited increased mRNA expression of *CNR2* and decreased expression of *FAAH1* and *NAAA*, similar to our observations. Unless the cytology examination, required to determine subclinical endometritis, was not performed in that study and animals had clinical signs, this parallel reinforces the importance of the ECS in maintaining endometrial health and managing inflammation. Their research also highlighted the role of the ECS components in modulating inflammatory responses, with elevated levels of pro-inflammatory cytokines such as TNF and IL1B associated with endometritis [25].

The role of *PTGDS* in our study deserves special attention. While its expression showed some inconsistency, there was a notable increase in *PTGDS* expression in cows with clinical endometritis at 4 weeks postpartum. PTGDS catalyzes the production of prostaglandin D2, which has been implicated in both pro- and anti-inflammatory processes [26]. The elevated *PTGDS* expression early in the postpartum period may reflect an initial pro-inflammatory response that could potentially shift toward an anti-inflammatory role as the condition progresses. This dual nature of PTGDS highlights the complex interplay between the ECS and other signaling systems in regulating endometrial inflammation.

Our observations regarding the expression of ECS-related genes in the context of fatty liver provide novel insights into the potential impact of metabolic disorders on endometrial health. The consistent downregulation of NAAA in cows with fatty liver, particularly at 7 weeks postpartum, suggests a potential link between hepatic lipid metabolism and endometrial ECS function. NAAA is involved in the degradation of N-acylethanolamines, including AEA, and its reduced expression could lead to altered levels of these bioactive lipids in the endometrium. This finding opens up new avenues for investigating the systemic effects of metabolic disorders on reproductive health in dairy cows.

The temporal changes observed in *CNR2* expression in cows with fatty liver, characterized by decreased expression at 4 weeks postpartum and a failure to increase at 7 weeks as seen in healthy cows, suggest that metabolic stress may interfere with the normal adaptive responses of the ECS during the postpartum period. This dysregulation could potentially contribute to impaired resolution of inflammation and delayed restoration of endometrial health in cows with fatty liver.

The insights gathered from our study have profound implications for managing endometrial health in dairy cows. The observed temporal dynamics of ECS gene expression suggest that the timing of the interventions may be critical for effectively mitigating postpartum reproductive disorders. Specifically, strategies targeting CB2 receptor activation or MAGL inhibition could yield optimal results when implemented at precise timepoints during the postpartum period. Future research should prioritize elucidating the causal relationships between ECS gene expression patterns and reproductive outcomes through comprehensive longitudinal studies. Additionally, expanding upon initial investigations [15,17] into the effects of dietary supplements, such as omega-3 fatty acids or conjugated linoleic acid, on ECS activity in the bovine endometrium could yield practical interventions for enhancing reproductive health. Furthermore, the exploration of selective CB2 receptor agonists or MAGL inhibitors as potential therapeutic agents for managing endometrial inflammation in dairy cows presents a promising avenue for translational research.

Comparative studies between bovine models and other species, including humans, could provide valuable insights into conserved mechanisms of ECS regulation in reproductive health. The similarities in CB2 receptor upregulation observed in our study and in women with endometrial inflammation [23] suggest potential for developing cross-species therapeutic strategies targeting the ECS.

While our study provides valuable insights into the expression of endocannabinoid system genes in the endometrium of postpartum dairy cows, it is important to acknowledge certain limitations. Our sample size, though considered adequate for genetic studies of this nature, may have limited our ability to detect statistically significant differences in some comparisons, particularly evident in cases wherein we observed trends that did not reach conventional levels of statistical significance. The high individual variability observed in some gene expression patterns, especially for MGLL, posed challenges in achieving statistical significance. Our focus on a specific set of genes within the endocannabinoid system, while carefully selected, may not capture all relevant pathways involved in the complex interplay between endometrial health, fatty liver and the endocannabinoid system. The limited number of cows without fatty liver (n = 8) restricted our ability to conduct a robust analysis of the interaction between endometritis and fatty liver. Additionally, our study’s design, with measurements at two specific time points postpartum, may not fully capture the dynamic and chronic nature of conditions like endometritis. Despite these limitations, our study provides valuable preliminary data and highlights important trends in gene expression that can inform future research directions, contributing to our understanding of the endocannabinoid system’s role in postpartum uterine health and metabolic status in dairy cows.

## 4. Materials and Methods

All experimental procedures in this study were designed and executed in strict adherence to the guidelines approved by The Local Ethics Committee for Animal Experiments in Olsztyn (Resolution No. 28/2020). This ensured full compliance with current European Union animal welfare laws, particularly the Directive 2010/63/EU of the European Parliament and Council on the protection of animals used for scientific purposes. This study was conducted on a herd of 200 Holstein Friesian dairy cattle located at the Teaching Station of the University of Warmia and Mazury in Olsztyn, Poland (53°77′ N, 20°47′ E).

### 4.1. Animal Husbandry Management

The animals were housed in a free-stall barn, meeting the requirements in the European animal welfare directives. The barn was equipped with rubber mats for comfort, manure scrapers and a ventilation system to maintain optimal air quality and temperature. Each cow had access to a minimum of 10 m^2^ of resting area and 25 cm of feed bunk space, ensuring comfortable living conditions and minimizing social stress.

The cows were provided with constant access to fresh, clean water through automated waterers, which were cleaned and inspected daily. The feeding regimen consisted of total mixed ration (TMR) delivered twice daily at 08:00 (±15 min) and 16:00 (±15 min), with feed available ad libitum. The TMR was mixed using a vertical mixer wagon to ensure homogeneity, with mixing time controlled to prevent over-processing of forage particles. Feed push-ups were performed every 2 h manually to stimulate feed intake and ensure consistent feed availability throughout the day.

The pre- and postpartum TMR compositions were designed to meet the nutritional requirements of transition and early lactation cows, respectively. The prepartum diet consisted primarily of 20 kg of silage, 8 kg of corn silage and 2.5 kg of specialized feed mixture for dry cows per cow. The protein–energy corrector in this diet contained 190 g/kg total protein and provided 1100 VEM (net energy for lactating cows). The postpartum diet was more energy-dense, comprising 25 kg of corn silage, 9 kg of silage and 3 kg of protein–energy corrector per cow. This corrector contained 380 g/kg total protein and provided 990 VEM. Both diets were supplemented with vitamin–mineral premixes tailored to each stage. The prepartum supplement was formulated with a negative DCAB (Dietary Cation–Anion Balance) of −5480, while the postpartum supplement included higher levels of vitamins and trace minerals, such as 900,000 U/kg of Vitamin A and 6000 mg/kg of Vitamin E.

**Figure 4:**
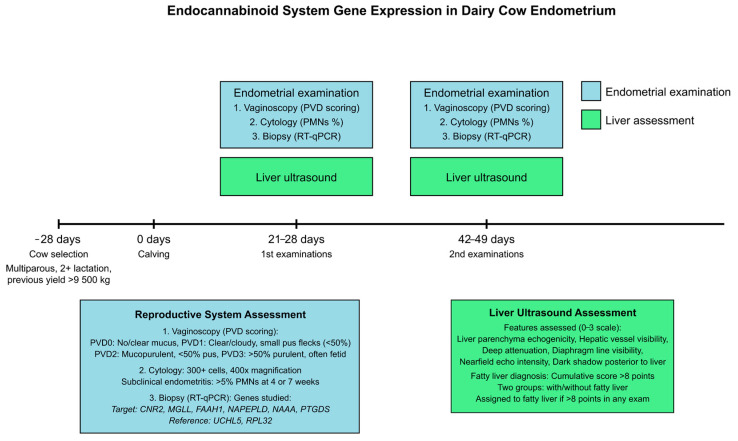
Experimental design and assessment protocol for studying endocannabinoid system gene expression in dairy cow endometrium. The timeline illustrates the study progression from cow selection (−28 days) through calving (0 days) to two sets of examinations at 21–28 and 42–49 days postpartum. Multiparous cows with previous lactation yield >9500 kg were selected. Each examination set included endometrial assessment (vaginoscopy, cytology and biopsy for RT-qPCR) and liver ultrasound. The reproductive system assessment involved PVD scoring, cytological evaluation for endometritis (threshold of >5% PMNs) and analysis of target genes (cannabinoid receptor 2 (*CNR2*), fatty acid amide hydrolase (*FAAH1*), N-acylethanolamine acid amidase (*NAAA*), N-acyl phosphatidylethanolamine phospholipase D (*NAPEPLD*), peptidyl arginine deiminase 4 (*PADI4*), prostaglandin D2 synthase (*PTGDS*) and monoacylglycerol lipase (*MGLL*)) with Ubiquitin C-Terminal Hydrolase L5 (*UCHL5*) and Ribosomal Protein L32 (*RPL32*) as references. Liver ultrasound assessed multiple features on a 0–3 scale, with fatty liver diagnosed at a cumulative score >8 points. This design enabled comprehensive investigation of endocannabinoid system gene expression in relation to both endometrial health and liver condition in postpartum dairy cows.

Three weeks prior to their expected calving date, cows were transferred to a prepartum pen. This pen was designed with extra bedding and increased space allowance (12 m^2^ per cow) to accommodate the physical changes associated with late pregnancy. The bedding material (straw) was replenished daily to maintain a dry, comfortable lying surface. The cows were monitored for signs of imminent calving, including udder enlargement, milk letdown and relaxation of the tail ligaments. This monitoring was performed every two hours by trained personnel, with increased frequency as parturition approached (Figure 4).

Upon detection of signs indicating imminent parturition, the animals were transferred to individual maternity pens, each measuring 16 m^2^ and bedded with clean, dry straw. Calving was supervised by trained personnel, with veterinary assistance available 24/7 if needed. Intervention during calving was minimized and only performed if necessary to ensure the well-being of the cow and calf.

Within 48 h of post-calving, cows were relocated to the postpartum pen. Postpartum cows were milked twice daily at 05:00 (±30 min) and 15:00 (±30 min) using a 2 × 6 parallel milking parlor (DeLaval, Sweden, Tumba). The milking routine included pre-dip ping with an iodine-based solution (0.5% iodine), thorough cleaning of teats with individual cloth towels, fore-stripping to check for abnormal milk, attachment of milking units within 90 s of first teat stimulation and post-dipping with a barrier teat dip containing 1% iodine. Milking units were automatically removed when milk flow decreased below 400 g/min. Milk yield was recorded at each milking using in-line milk meters, and milk samples were collected monthly for composition analysis (fat, protein, lactose and somatic cell count) using a Fossomatic 7 analyzer (Foss, Denmark, Hillerød).

Over a six-month period from 1 July to 31 December 2020, all cows within three weeks of their expected calving date were closely monitored. Health assessments were conducted daily, including evaluation of appetite, rumination (visually), fecal consistency and general demeanor. Rectal temperature was measured daily for the first 10 days postpartum, and any cow with a temperature above 39.5 °C was subjected to a thorough clinical examination. Body condition scoring (BCS) was performed weekly using a 1–5 scale with 0.25-point increments, as described by Ferguson et al. [27]. All health events, treatments and management interventions were recorded in individual cow health cards and in a computerized herd management system (DeLaval, Sweden, Tumba).

### 4.2. Inclusion and Exclusion Criteria

Cows were selected for inclusion in this study based on criteria evaluated up to one week before the expected calving date. Eligible cows were multiparous, meaning they were entering their second or subsequent lactation, with a previous lactation milk yield exceeding 9500 kg (305-day mature equivalent). Only cows that exhibited overall good health, with no signs of clinical disease and no history of chronic health issues, were considered eligible. However, cows initially meeting these inclusion criteria were subsequently excluded from the study if they developed clinical diseases requiring medication or surgical intervention, experienced complications during calving—such as dystocia or retained placenta—or required antibiotic or anti-inflammatory treatments during the period from one week before calving through to the end of the study (Figure 4).

From day 21 postpartum, enrolled cows underwent weekly health evaluations, including physical examinations, rectal temperature measurements and assessments of vaginal discharge. Additionally, ultrasound liver evaluations, endometrial cytology and endometrial biopsy were conducted during two specific periods: between days 21 and 28 postpartum and again between days 42 and 49 postpartum. These time points were strategically chosen to capture the dynamic changes in endometrial health and gene expression during the postpartum period. Moreover, blood samples were collected weekly for hematology and biochemistry analyses from one week before calving until nine weeks postpartum, providing data on the physiological and metabolic changes occurring during this period (Figure 4).

### 4.3. Uterine and Liver Health Assessment

To evaluate the health status of the reproductive tract vaginoscopy and evaluation of vaginal mucus and endometrial cytology were performed (Figure 4).

Vaginoscopy was conducted on all cows between days 21 and 28 postpartum and again between days 42 and 49 postpartum. The procedure was carried out using a sterilized KRUUSE Vaginal Speculum (35 cm length, 5 cm width). Prior to the examination, each cow was properly restrained in a comfortable standing position within a designated examination area. The perineal area was thoroughly cleaned and disinfected with alcohol. The speculum was lubricated with a sterile gel to minimize discomfort during insertion. It was gently introduced into the vagina at a slight upward angle to avoid entering the urethral opening. Once fully inserted, the speculum was opened to allow for visualization of the vaginal walls and cervix.

The appearance of vaginal mucus was carefully assessed and categorized according to the following purulent vaginal discharge (PVD) scoring system: PVD0 indicated no mucus or clear, translucent mucus; PVD1 involved clear or cloudy mucus with small flecks of pus (<50% pus); PVD2 was mucopurulent discharge containing less than 50% pus in mucus; and PVD3 was discharge containing >50% purulent material, often fetid. The entire examination was performed under good lighting conditions provided by a handheld LED light source, with observations recorded immediately on a standardized form. After each use, the speculum was thoroughly cleaned with soap and water, disinfected with a 2% chlorhexidine solution and sterilized with alcohol before the next use.

Following vaginoscopy, endometrial cytological samples were collected using the cytobrush technique. A sterile cytobrush (Cervical Brush, Zarys International Group, Poland, Warszawa) was mounted on a stainless-steel rod and inserted into a sanitized stainless-steel catheter (50 cm length, 6 mm external diameter). The assembled device was protected with a disposable plastic sleeve to prevent contamination during passage through the vagina. The vulva was cleaned and disinfected again, and the cytobrush apparatus was carefully introduced into the vagina, guided by rectal palpation. Once the external cervical os was reached, the catheter was gently passed through the cervix. Upon complete passage through the cervix and entry into the uterine body, the plastic sleeve was punctured. The cytobrush was then extended approximately 1 cm beyond the catheter tip. The exposed brush was rotated clockwise against the endometrium of the right uterine horn, making a full 360° turn to ensure adequate sample collection. The brush was then retracted into the catheter, and the entire apparatus was gently withdrawn from the reproductive tract.

Immediately after collection, the cytobrush was rolled onto two clean glass microscope slides, creating duplicate smears. The slides were air-dried and then stained using the Hemavet rapid staining kit (Hemavet, Kolchem, Poland, Kraków) according to the manufacturer’s instructions. This kit employs a modified Wright-Giemsa stain, which allows for clear differentiation of cell types. Cytological evaluation was performed by a trained veterinary cytologist who was blinded to the clinical status of the cows. A minimum of 300 cells were counted on each slide under a light microscope (Olympus BX43, Olympus Corporation, Japan, Tokio) at 400× magnification. The percentage of polymorphonuclear neutrophils (PMNs) relative to epithelial cells was calculated. Subclinical endometritis was diagnosed when the proportion of PMNs exceeded 5% at 4 or 7 weeks postpartum, in the absence of clinical signs. This threshold was based on previously published studies and has been shown to be associated with reduced reproductive performance in dairy cows [28].

To assess liver health status, ultrasound examinations were performed twice for each cow: first between 21 and 28 and then between 42 and 49 days postpartum and a portable 4 Vet Slim ultrasound device equipped with a 3.5 MHz convex probe (Dramiński, Olsztyn, Poland) was used. The area between the 11th and 13th intercostal spaces on the right side of the cow was clipped and cleaned with 70% isopropyl alcohol. Ultrasound gel was applied to ensure good contact and image quality. The probe was placed in intercostal spaces and moved dorsoventrally to visualize different areas of the liver. Ultrasound videos were recorded and saved in DICOM format for subsequent measurements.

The captured images were analyzed using MicroDicom software (MicroDicom Ltd., Sofia, Bulgaria, version 3.1.4) by a veterinary radiologist with over 5 years of experience in bovine ultrasonography, who was blinded to the clinical status of the cows. The ultrasound images were evaluated for signs of fatty infiltration in the liver, following the methodology outlined by Komeilian et al. [29]. This involved assessing the following features: liver parenchyma echogenicity, hepatic vessel visibility, presence of deep attenuation, diaphragm line visibility, nearfield echo intensity and presence of a dark shadow posterior to the liver. Each feature was scored on a scale of 0–3, with higher scores indicating more severe fatty infiltration. A cumulative score was calculated and cows were classified as having fatty liver if their score exceeded a predetermined threshold of 8 points, based on previous validation studies [30]. For the purposes of this study, we did not distinguish between different stages of fatty liver severity. Based on the liver image analysis results, the cows were divided into two groups: those without fatty liver and those with fatty liver. Animals were assigned to the fatty liver group if they exceeded the threshold of 8 points in at least one examination.

### 4.4. Group Assignment

A total of 22 cows (mean parity 3.01 ± 1.31; mean ± SD) met the inclusion criteria described in Section 4.2 and were enrolled in the study. These animals were subjected to an evaluation for the presence of clinical and subclinical endometritis, as well as fatty liver, as described in Section 4.3 (Figure 4).

The cows were categorized into several groups based on their health status at different time points. The first analysis included the following groups: at 4 weeks postpartum, healthy cows (H4, n = 11), subclinical endometritis (SE4, n = 6) and clinical endometritis (CE4, n = 5); at 7 weeks postpartum, healthy cows (H7, n = 14), subclinical endometritis (SE7, n = 5) and clinical endometritis (CE7, n = 3). For the second analysis, SE and CE cows were considered together, so the groups were as follows: at 4 weeks postpartum, healthy cows (H4, n = 11) and combined endometritis (E4, n = 11); at 7 weeks postpartum, healthy cows (H7, n = 14) and combined endometritis (E7, n = 8). A third grouping was based on the presence of fatty liver: at 4 weeks postpartum, without fatty liver (HL4, n = 8) and with fatty liver (FL4, n = 14); at 7 weeks postpartum, without fatty liver (HL7, n = 8) and with fatty liver (FL7, n = 14). This multi-faceted grouping strategy allowed for a comprehensive analysis of gene expression data across different health conditions and postpartum stages.

### 4.5. Endometrial Biopsy and RNA Extraction

Endometrial biopsies were collected at 4 and 7 weeks postpartum, resulting in a total of 44 biopsy samples for analysis. The sample size was determined based on previous studies investigating gene expression in bovine endometrium, with consideration given to the expected variability in gene expression and the desired statistical power to detect biologically relevant differences (Figure 4).

Endometrial biopsy samples were collected using sterile, disposable Kevorkian’s uterine biopsy forceps (Hauptner Herberholz, Solingen, Germany). The perineal area was thoroughly cleaned and disinfected with alcohol. The biopsy forceps, sheathed in a sanitized plastic sleeve, were introduced into the vagina and guided through the cervix via rectal manipulation. Upon reaching the uterine lumen, the plastic sleeve was retracted, and the forceps were advanced to the dorsal wall of the uterine horn. The jaws of the forceps were opened and pressed gently against the endometrium. A small piece of tissue (approximately 10–20 mg) was obtained by closing the jaws and simultaneously rotating the instrument. The forceps were then carefully withdrawn from the reproductive tract. The biopsy sample was immediately removed from the forceps using a sterile needle and placed in a labeled cryotube and immediately frozen in liquid nitrogen. In the laboratory, samples were stored at −80 °C until further processing.

Total RNA isolation was performed after completing the field portion of the experiment and after all samples had been collected and stored at −80 °C. The extraction process followed the PureLink RNA Mini Kit protocol (Thermo Fisher Scientific, Waltham, MA, USA) with some modifications to optimize yield and quality. Samples were removed from the −80 °C freezer and immediately placed in a container with crushed ice to prevent RNA degradation. Each frozen tissue sample (10–20 mg) was placed in a 2 mL microcentrifuge tube containing a 5 mm stainless-steel bead (Qiagen, Hilden, Germany) and 600 µL of lysis buffer supplemented with 1% 2-mercaptoethanol (Sigma-Aldrich, St. Louis, MO, USA). Samples were homogenized using a TissueLyser LT (Qiagen, Hilden, Germany) for 2 min at 50 Hz. The homogenized lysate was centrifuged at 12,000× *g* for 2 min at 4 °C, and the supernatant was transferred to a new tube. An equal volume of 70% ethanol was added to the cleared lysate and mixed thoroughly. The mixture was transferred to a spin cartridge and centrifuged at 12,000× *g* for 15 s at room temperature. The flow-through was discarded. The spin cartridge was washed with Wash Buffer I and then twice with Wash Buffer II as per the manufacturer’s instructions. After the final wash, the spin cartridge was centrifuged at 12,000× *g* for 2 min to remove any residual wash buffer. RNA was eluted by adding 50 µL of RNase-free water to the center of the spin cartridge, incubating for 1 min at room temperature and then centrifuging at 12,000× *g* for 2 min.

The quantity and purity of the extracted RNA were assessed using a NanoDrop ND1000 spectrophotometer (Thermo Scientific, Waltham, MA, USA). The A260/A280 and A260/A230 ratios were measured to assess protein and organic solvent contamination, respectively. Samples with A260/A280 ratios between 1.8 and 2.1 and A260/A230 ratios above 1.8 were considered acceptable purity. The RNA integrity number (RIN) was determined using capillary electrophoresis with an Agilent 2100 Bioanalyzer (Agilent Technologies, Santa Clara, CA, USA) and the Agilent RNA 6000 Nano Kit (Agilent Technologies, Santa Clara, CA, USA), following the manufacturer’s guidelines. A minimum RIN value of 8 was required for a sample to be included in further analysis. The quality and purity of RNA samples did not significantly differ between groups and collection times. To ensure RNA stability and prevent degradation, aliquots of the RNA samples were stored at −80 °C until reverse transcription. Each aliquot was thawed only once to avoid freeze–thaw cycles that could compromise RNA integrity.

### 4.6. cDNA Synthesis and RT-qPCR Analysis

Reverse transcription was performed using the Transcriptor First Strand cDNA Synthesis Kit (Roche, Basel, Switzerland) following the manufacturer’s instructions. For each reaction, 1000 ng of total RNA was used. The reaction mixture consisted of 1 µL of anchored-oligo(dT)18 primer (50 pmol/µL), 2 µL of random hexamer primer (600 pmol/µL), 4 µL of 5× Transcriptor Reverse Transcriptase Reaction Buffer, 0.5 µL of Protector RNase Inhibitor (40 U/µL), 2 µL of Deoxynucleotide Mix (10 mM each) and 0.5 µL of Transcriptor Reverse Transcriptase (20 U/µL), with PCR-grade water added to reach a final volume of 20 µL. The mixture was gently mixed and briefly centrifuged. Reverse transcription was carried out in a thermal cycler with a heated lid using the following program: 25 °C for 10 min (primer annealing), 55 °C for 30 min (cDNA synthesis) and 85 °C for 5 min (enzyme inactivation). The reaction was immediately stopped by placing the tube on ice. The resulting cDNA was diluted 100-fold with nuclease-free water and stored at −20 °C until use in qRT-PCR analysis. To control potential genomic DNA contamination, a no-reverse transcriptase (RT-) control was included for each RNA sample, where the reverse transcriptase enzyme was replaced with an equal volume of PCR-grade water.

The qRT-PCR experiments were conducted on a LightCycler LC 480 II system (Roche, Basel, Switzerland) using LightCycler 480 Multiwell Plates 96 (Roche, Basel, Switzerland). Each reaction was set up in a total volume of 20 µL containing 5 µL of the 100-fold diluted cDNA, 10 pmol of each specific primer (forward and reverse), 3 µL of PCR-grade water and 10 µL of LightCycler 480 SYBR Green I Master mix (Roche, Basel, Switzerland). Primers for all target genes and reference genes were designed using Primer-BLAST (available at https://www.ncbi.nlm.nih.gov/tools/primer-blast/ (accessed on 2 September 2022)), with the following criteria: amplicon size of 60–200 bp, primer length of 18–25 bp, GC content of 40–60%, melting temperature (Tm) of 58–62 °C with a maximum 2 °C difference between primer pairs and spanning an exon–exon junction to avoid amplification of genomic DNA. The sequences of all primers used are provided in Table 1. *UCHL5* and *RPL32* were used as reference genes for normalizing the RT-qPCR data, as they showed stable expression across all experimental conditions in preliminary analyses.

Each reaction was performed in technical duplicate, and a negative control (no template control, NTC) which substituted PCR-grade water for cDNA, was included and run in triplicate on each plate to ensure result reliability and check for potential contamination. The qPCR cycling conditions were as follows: initial denaturation at 95 °C for 5 min, followed by 45 cycles of denaturation at 95°C for 10 s, annealing at 60 °C for 15 s and extension at 72 °C for 20 s. A melting curve analysis was performed from 65 °C to 97 °C with a heating rate of 0.11 °C/s to confirm the specificity of the amplification products.

For each gene target analyzed, PCR reaction efficiency and error margins were determined using a 6-point standard curve generated from 4-fold serial dilutions of pooled cDNA. Only primers with efficiencies between 95% and 105% and error values less than 0.1 were used in the study. Quantification cycle (Cq) values were obtained using the LightCycler 480 SW 1.5 software (Roche, Basel, Switzerland), employing the software’s default settings and the second derivative maximum method for accurate quantification of target cDNA in the samples.

### 4.7. Statistical Analyses

All data were collected and initially organized in Microsoft Excel 365 (Microsoft Corporation, Redmond, WA, USA). Descriptive statistics, including mean values and standard deviations for each variable at different time points, were calculated using Python 3.10.0 (Python Software Foundation, Wilmington, DE, USA) and R 4.3.1 (R Core Team, Vienna, Austria). The Shapiro–Wilk test was used to evaluate the normality of the data distribution, and Levene’s test was applied to verify the homogeneity of variances across groups. Due to the non-parametric nature of the data, as evidenced by these tests, appropriate non-parametric statistical methods were used for analysis.

The Mann–Whitney U test was employed to compare gene expression levels between groups (healthy vs. endometritis, and with vs. without fatty liver) on specific days during the study. For comparisons involving more than two groups (e.g., healthy vs. subclinical endometritis vs. clinical endometritis), the Kruskal–Wallis test was used, followed by Dunn’s post hoc test for pairwise comparisons when significant differences were detected. The Benjamini–Hochberg procedure was applied to control the false discovery rate when multiple comparisons were made, with an adjusted *p*-value < 0.05, considered statistically significant.

Spearman’s rank correlation coefficient was used to assess the correlation between gene expressions and between gene expression and other continuous variables (e.g., milk yield, days postpartum). The strength of the correlations was interpreted as follows: 0.00–0.19 “very weak”, 0.20–0.39 “weak”, 0.40–0.59 “moderate”, 0.60–0.79 “strong” and 0.80–1.0 “very strong”. *p*-values for correlation coefficients were adjusted using the Benjamini–Hochberg method to account for multiple testing.

Gene expression levels were quantitatively analyzed using RT-qPCR and the 2^−ΔΔCt^ method, as described by Schmittgen and Livak [31], with results rounded to two decimal places and expressed as fold changes with standard error of the mean (SEM). The healthy cow group at 4 weeks postpartum (H4) was used as the calibrator group for all comparisons. The fold change (FC) for each gene was calculated using the following formula:

$$FC=2^{-\Delta \Delta Ct},\mathrm{where}\;\Delta \Delta Ct={\left(Ct_{\mathrm{target}}-Ct_{\mathrm{reference}}\right)}_{\mathrm{sample}}-{\left(Ct_{\mathrm{target}}-Ct_{\mathrm{reference}}\right)}_{\mathrm{calibrator}}$$

The SEM for each fold change was calculated using the delta method, which takes into account the variability in both the target and reference gene measurements.

Matplotlib (version 3.5.1), a standard tool for data visualization in Python, was used to generate line and bar graphs to visually represent the gene expression data and other relevant parameters. Error bars on graphs represent the SEM.

Power analysis was conducted post hoc using G*Power (version 3.1.9.7, Heinrich-Heine-Universität Düsseldorf, Germany, Düsseldorf) to determine the achieved power given the sample size and observed effect sizes. For the main comparisons between healthy and endometritis groups, with an alpha level of 0.05 and the observed effect sizes, the achieved power ranged from 0.65 to 0.85 for detecting large effects (Cohen’s d > 0.8).

## 5. Conclusions

This study provides compelling evidence for the dynamic regulation of the endocannabinoid system (ECS) in the postpartum bovine endometrium and its potential involvement in uterine health and recovery. The observed patterns of gene expression, particularly the upregulation of *MGLL* in endometritis and the downregulation of *NAAA* in fatty liver, suggest that the ECS may play a crucial role in modulating inflammatory responses and lipid metabolism in the postpartum uterus. These findings not only enhance our understanding of the molecular mechanisms underlying postpartum uterine health in dairy cows but also open new avenues for potential therapeutic interventions. The temporal changes in gene expression highlight the importance of considering the dynamic postpartum environment when studying endometrial health. Furthermore, the apparent link between ECS dysregulation and metabolic disorders like fatty liver underscores the complex interplay between systemic metabolism and reproductive health in dairy cows. While further research is needed to elucidate the precise mechanisms and functional consequences of these gene expression changes, this study lays a strong foundation for future investigations into the role of the ECS in bovine reproductive health.

## Figures and Tables

**Table 1:** Oligonucleotide sequences used for quantitative real-time polymerase chain reaction (qRT-PCR) and characteristics of the resulting amplified products.

Gene Symbol	Primer Sequences	Annealing Temp.	T_m_	Amplicon Size	E	Error
	(F—Forward/R—Reverse) (5′-3′)	(°C)	(°C)	bp	10^−1/slope^	
*CNR2*	F-5′TCTTCGCCGGCATCATCTAC3′/ R-5′CATCCGGGCTATTCCAGACA3′	60	86.53	110	1.99	0.0233
*FAAH1*	F-5′TTCCTGCCAAGCAACATACCT3′/ R-5′CACGAAATCACCTTTGAAGTTCTG3′	60	85.10	105	2.04	0.0056
*NAAA*	F-5′CAGCACTACGACCGGGACTT3′/ R-5′CCGGGACGACTTTTCTGATC3′	60	84.05	100	1.98	0.0058
*NAPEPLD*	F-5′AGAGATCACAGCAGCGTTCCAT3′/ R-5′ACTCCAGCTTCTTCAGGGTCATC3′	60	79.85	95	1.99	0.0210
*PADI4*	F-5′CTACTACGCCTACCACGTCC3′/ R-5′TGCCACCACTTGAAGGAGAA3′	60	87.62	84	1.96	0.0094
*PTGDS*	F-5′TGAGACGCGGACCTTACTG3′/ R-5′CTGGGAGCGGCTGTAGAG3′	60	89.91	193	2.02	0.0257
*MGLL*	F-5′GCAACCAGCTGCTCAACAC3′/ R-5′AGCGTCTTGTCCTGGCTCTT3′	60	90.57	154	1.95	0.0120
*RPL32*	F-5′AAAGAGGACCAAGAAGTTCATTAGG3′/ R-5′CGCCAGTTCCGCTTGATTT3′	60	77.81	66	1.95	0.0118
*UCHL5*	F-5′ ACAAAGACAACTTGCTGAGGAACCC3′/ R-5′ GGCAACCTCTGACTGAATAGCACTT3′	60	78.80	79	1.97	0.0061

E−PCR efficiency calculated using formula E = 10^−1/slope^, error value—mean squared error of the single data point fit to the regression line, Tm—melting temperature curve peak value.

## Data Availability

The datasets used and analyzed during the current study are available from the corresponding author upon reasonable request.
